# Knowledge and use of e-cigarettes among nursing students: results from a cross-sectional survey in north-eastern Italy

**DOI:** 10.1186/s12889-019-7250-y

**Published:** 2019-07-22

**Authors:** Federica Canzan, Eliana Finocchio, Francesca Moretti, Silvia Vincenzi, Alex Tchepnou-Kouaya, Oliva Marognolli, Albino Poli, Giuseppe Verlato

**Affiliations:** 1 School of Nursing, University of Verona, Verona, Italy; 2 Unit of Epidemiology & Medical Statistics, Department of Diagnostics and Public Health, University of Verona, Verona, Italy; 3 Unit of Hygiene, Department of Diagnostics and Public Health, University of Verona, Verona, Italy

**Keywords:** E-cigarette, Nursing students, Dual use, Susceptibility to smoking, Smoking cessation

## Abstract

**Background:**

Data on electronic cigarette (e-cigarette) use among health professional students, who can play a central role in promoting healthy habits and smoking cessation, are sparse. Moreover, the association between e-cigarettes and smoking habits is still debated. The present study aimed to investigate the diffusion of e-cigarette use among nursing students in north-eastern Italy and explore its association with tobacco smoking.

**Methods:**

In 2015, a questionnaire focused on e-cigarette use and tobacco smoking habits was anonymously administered to 2020 students attending nursing courses held by Verona University in 5 different centres. Of these students, 1463 (72.4%) answered the questionnaire. The influence of e-cigarette ever use on both tobacco smoking initiation in all subjects and smoking cessation among ever smokers was investigated by multivariable logistic models.

**Results:**

Most responders were female (77.1%), and the mean (SD) age was 23.2 (4.2) years. Nearly all students (94.7%) had heard about e-cigarettes. Approximately one-third (30.3, 95% CI 27.9–32.7%) had ever used e-cigarettes, but only 2.1% (1.5–3.0%) had used e-cigarettes in the last month. Very few (2.1%) of those responders who had never used e-cigarettes were willing to try them. Prevalence values were much higher for tobacco smoking: 40.9% of responders reported being current tobacco smokers, and 10.1% reported being past smokers. Ever use and current use of e-cigarettes were reported by 57.2 and 4.4% of current tobacco smokers and by 12.0 and 0.6% of never or past smokers, respectively (*p* < 0.001). In multivariable analysis, students who ever used e-cigarettes had 13 times greater odds of being an ever tobacco smoker than never users, whereas they had three times lower odds of being a former smoker.

Only 26 students were currently using both electronic and tobacco cigarettes, and most declared that they used e-cigarettes to stop or reduce tobacco smoking. Of note, only three students reported that they had completely stopped smoking thanks to e-cigarette use.

**Conclusion:**

Use of e-cigarettes seemed to be rather rare among Italian nursing students and was mainly restricted to current smokers. E-cigarette use was not associated with smoking cessation in nursing students.

**Electronic supplementary material:**

The online version of this article (10.1186/s12889-019-7250-y) contains supplementary material, which is available to authorized users.

## Background

Electronic cigarette (e-cigarette) use has been rapidly spreading around the world [1–3]. Indeed, the idea that e-cigarette use is safer than tobacco smoking is very common in the world population, as conditioned by manufacturers’ advertisements.

The scientific community has been dividing about e-cigarette use. The Royal College of Physicians stated that e-cigarettes represent a “viable harm-reduction option” with respect to tobacco smoking and that “the hazard to health arising from long-term vapour inhalation from the e-cigarettes available today is unlikely to exceed 5% of the harm from smoking tobacco” [4]. Accordingly, a simulation study predicted that if common cigarettes were replaced by e-cigarettes over the next 10 years, 1.6 to 6.6 million premature deaths would be avoided in the United States in the current century [5].

In contrast, the European Respiratory Society (ERS) stated that there was no evidence that e-cigarettes could be safer than tobacco in the long term, although it was acknowledged that potentially toxic chemicals were fewer and less concentrated in e-cigarette aerosols than in conventional cigarettes [6]. Moreover, according to the same ERS report, “there is not enough evidence to support that e-cigarettes are an aid to smoking cessation”, and “there is conflicting data that use of e-cigarettes results in a renormalisation of smoking behaviour or for the gateway hypothesis” [6]. Other studies supported the notion that e-cigarettes could not be considered harmless, despite being less noxious than conventional tobacco cigarettes [7], because they also contained several unstudied and unregulated compounds [8].

Moreover, while trends in the tobacco smoking epidemic have been extensively studied [9, 10], the more recent trend in e-cigarette use is less established. In the Eurobarometer survey, performed on the European Union population in 2012, only 7.2% of people aged 15 years or older reported having ever used e-cigarettes; however, ever use was higher among students and people younger than 35 years [11]. Accordingly, in a cross-sectional study on the Italian general population [12], the use of the electronic cigarette decreased with advancing age and was the highest among people aged 15–24 years: in this age range, 94.8% of those interviewed had heard about e-cigarettes, 9.2% had tried them, and 2.4% were regularly users. In the United States, e-cigarettes were the most commonly used tobacco product among middle (5.3%) and high (16.0%) school students in 2015 [13].

From a public health perspective, the major question dealing with e-cigarette use is its relation to tobacco smoking: does e-cigarette use decrease smoking consumption and promote smoking cessation, or does it represent a gateway to nicotine addiction and consequently to tobacco smoking initiation? A Cochrane Review, updated in 2016, concluded that nicotine e-cigarettes helped smokers stop smoking in the long term compared with placebo e-cigarettes, but the evidence was rated low under the GRADE system [14]. An opposite conclusion was achieved by a meta-analysis of 38 studies focused on e-cigarettes and smoking cessation in real-world and clinical settings: the odds of quitting traditional cigarettes were 28% lower in those who used e-cigarettes than in those who did not [15].

Among adolescents, e-cigarettes seem to be a gateway to tobacco smoking. A recent meta-analysis on people aged 14–30 years found that the risk of tobacco smoking was 3–4 times higher in e-cigarettes ever users than in never users [16]. E-cigarettes seem to play a similar role in tobacco smoking initiation in adults. Indeed, in an Italian longitudinal study on people aged 30–75 years [17], exclusive e-cigarette users were much more likely to have switched to tobacco smoking after 2 years of follow-up (38.9%) than to have quit whatever type of smoking (18.8%). Conversely, only 26% of dual users had quit tobacco smoking, whereas 57.4% had started smoking only tobacco.

The increase in e-cigarette use, particularly among teenagers and young adults [11–13], as well as the limited evidence regarding the safety profile of e-cigarettes [7], highlight the urgent need for reinforcing education and awareness of e-cigarette use, starting from school and university educational programmes. In this respect, the smoking attitudes and behaviours of nursing students are particularly important because, as future health professionals, they will play a central role in promoting healthy habits and counselling their patients about smoking cessation [18]. Moreover, understanding nursing students’ smoking habits might be valuable for the future development of tobacco control activities promoted by the university. However, there is scarce knowledge on e-cigarette use among nursing students in Europe. A recent study on French military nursing students found a high prevalence of both ever and current e-cigarette use (36 and 25%, respectively) [19]. Moreover, studies on medical/health professional students found a large variability in e-cigarette current use, with the proportion of current vapers ranging from 1.6% [20] to 20.6% [21]. To date, there are no data on e-cigarette use among Italian nursing students. The present study aimed to i) investigate the diffusion of e-cigarette use among nursing students in north-eastern Italy and ii) explore its association with tobacco smoking.

## Methods

This cross-sectional survey was administered in September 2015 to undergraduate students attending the Nursing School of Verona University at 5 centres in north-eastern Italy. Three centres are located in the plane (Verona, Vicenza, Legnago), and two are located in a valley inside the Alps (Trento, Bolzano).

Students were anonymously administered an Italian modified version of the French questionnaire ETINCEL-OFDT (“Enquête téléphonique pour l’information sur la cigarette électronique” of the “Observatoire français des drogues et des toxicomanies”) [22, 23], which focused on e-cigarette use and tobacco smoking habits. The original version of the questionnaire was translated into Italian by a native Italian speaker and back-translated by a native French speaker. An expert panel, which was composed of two nursing teachers and one sociologist, identified and resolved the inadequate expressions/concepts of the translation. Questions on smoking habits among relatives and roommates were added to the Italian version of the questionnaire. The English version of the questionnaire used is available as Additional file 1.

All students attending the Nursing School of Verona University were fully informed about the purpose of the research and asked to participate in the survey during the laboratory practice, which was mandatory to complete the nursing course. Students were administered an anonymous questionnaire and were given approximately 15 min to answer it. No incentive was provided to complete the questionnaire.

Smoking status was defined by asking participants if they currently smoked and how frequently. Smokers were classified as “regular smokers” if they reported smoking either daily or several times a week and “occasional smokers” if they smoked less frequently, i.e., “once a week”, “two-three times a month” or “once a month”. “Past smokers” were subjects who had smoked during their lifetime but did not smoke at the time of the survey. Subjects who had never smoked or had just tried smoking were considered “never smokers”.

Participants were also asked about their awareness of e-cigarettes, their past and present use of e-cigarettes, and their intention to try e-cigarettes in the future. Participants currently using both electronic and conventional cigarettes were classified as “dual users”. The participants were asked to select their main reasons for using e-cigarettes from a list of several options (including “to quit smoking”, “to reduce cigarette consumption”, and “to reduce harmful health effects”). Finally, former smokers were asked if e-cigarette consumption had helped them quit smoking.

### Statistical analysis

The relation between tobacco smoking and e-cigarette use was evaluated by Fisher’s exact test or chi-square test for trend. The influence of tobacco smoking on e-cigarette ever use was further investigated by a multivariable logistic model, controlling for centre, gender, university class, family history of smoking habits, and smoking habits among current housemates.

The effects of e-cigarette ever use on ever smoking or former smoking were investigated by applying two separate logistic regression models that estimated 1) the odds ratios (ORs) of being an ever smoker in the whole sample and 2) the ORs of being an ex-smoker among ever smokers. Centre, gender, university class, family history of smoking habits, and smoking habits among current housemates were also introduced in the logistic models as potential confounders. Standard errors were adjusted for intra-centre correlation. Statistical significance was set at *p* < 0.05, and the significance of the ORs was computed by the Wald test. Stata (R) software (Texas, USA, version 14.0) was used for statistical analysis.

## Results

Among the 2016 students attending the courses, 1463 (72.6%) answered and returned the questionnaire. Most responders were female (1108/1438 = 77.1%), and the mean (SD) age was 23.2 (4.2) years. A greater percentage of non-responders than responders were students in the first class; hence, responders were slightly older. The response rate was the highest in Vicenza and the lowest in Verona, while it was not affected by gender (Table 1).

**Table 1:** Demographic characteristics of Verona nursing students who responded or did not respond to the questionnaire on e-cigarette use and tobacco smoking

	Responders (*n* = 1463)	Non-responders (*n* = 557)	*p*-value
Gender (women)	1108 (77.1%)	468 (81.0%)	0.056
Age, in years (mean (SD))	23.2 (4.2)	19.2 (3.0)	< 0.001
Class			< 0.001
1st	452 (30.9%)	282 (50.8%)	
2nd	474 (32.4%)	165 (29.7%)	
3rd	535 (36.6%)	108 (19.5%)	
Centre			< 0.001
Verona	566 (38.7%)	296 (53.5%)	
Vicenza	238 (16.3%)	45 (8.1%)	
Legnago	205 (14.0%)	74 (13.4%)	
Trento^a^	177 (12.1%)	57 (10.3%)	
Bolzano	277 (18.9%)	81 (14.6%)	

*p-*values were computed by Fisher’s exact test for gender and centre, by chi-square test for trend for class, and by t test for age

^a^In Trento, the anonymous questionnaire was not administered to nursing students in the 1st class

Nearly all responders (1379/1456 = 94.7%) had an awareness of e-cigarettes. Approximately one-third (442/1460 = 30.3, 95% CI 27.9–32.7%) of the responders had ever used e-cigarettes, but only 2.1% (31/1452, 95% CI 1.5–3.0%) of them declared themselves to be “current e-cigarette users”, i.e., to have been using e-cigarettes during the month before completing the survey. Very few (21/1015 = 2.1%) of those responders who had never tried e-cigarettes were willing to try them.

Ever and current use of e-cigarettes largely differed by gender: nearly half of the male students who responded (43.8, 95% CI 38.3–49.3%) had ever used e-cigarettes, while only 26.3% (95% CI 23.7–29.0%) of the female students who responded had. Likewise, the prevalence of e-cigarette current use was more than doubled in males (3.7, 95% CI 1.9–6.4%) with respect to that in females (1.7, 95% CI 1.0–2.7%). Ever or current use was not significantly affected by either class or centre (Table 2). Notably, ever use tended to be more common in centres located in the plane than in centres in Alpine valleys (*p* = 0.065). Age was similar in people who had tried or had never tried e-cigarettes (23.2 (4.4) vs 23.1 (3.8) years; *p* = 0.125) as well as among current users and other people (23.2 (4.2) vs 24.1 (5.1) years; *p* = 0.384). Ever use of e-cigarettes was more frequently reported by students whose relatives/housemates smoked either e-cigarettes or tobacco.

**Table 2:** Ever and current e-cigarette use among nursing students of Verona University by demographic characteristics

	E-cigarette ever users (*n* = 442)	*p*-value	E-cigarette current users (*n* = 31)	*p-*value
Gender^a^		**< 0.001**		**0.048**
Men	**144/329 (43.8)**		**12/324 (3.7)**	
Women	**291/1107 (26.3)**		**19/1104 (1.7)**	
Class^a^		0.738		0.398
1st	140/451 (31.0)		10/447 (2.2)	
2nd	141/474 (29.7)		13/471 (2.8)	
3rd	160/533 (30.0)		8/532 (1.5)	
Centre		0.065		0.782
Verona	191/579 (33.0)		10/574 (1.7)	
Vicenza	77/238 (32.4)		7/238 (2.9)	
Legnago	63/199 (31.7)		4/198 (2.02)	
Trento	42/177 (23.7)		3/176 (1.7)	
Bolzano	69/267 (25.8)		7/266 (2.6)	

Numbers of ever or current users of e-cigarettes were reported with percent frequencies in parentheses. *p*-values were computed by Fisher’s exact test for sex and centre and by chi-square test for trend for class. Significant results are highlighted in bold

^a^Information on gender and university class was not reported by 25 and 2 students, respectively

Prevalence values were much higher for tobacco smoking: 591 out of 1444 responders (40.9, 95% CI 38.4–43.5%) reported that they were currently smokers. In particular, 407 responders (28.2%) were classified as regular smokers, as they declared that they had been smoking daily or several times per week for the previous month, while 169 responders (11.7%) were classified as occasional smokers, as they reported that they had been smoking once a week or less frequently. Never smokers were nearly half of the studied population (683/1444 = 47.3%), while past smokers were only 10.1% (146/1444). Fifteen smokers who had given no information about their smoking frequency and 24 non-smokers who had given no information about their past smoking history were excluded from multivariable analyses. Regarding e-cigarette use, tobacco smoking habits were more prevalent among males than females and among students whose relatives/housemates smoked either tobacco or e-cigarettes. Tobacco smoking habits were not significantly affected by either class or centre (Table 3).

**Table 3:** Tobacco smoking among nursing students of Verona University by demographic characteristics

	n	Past smokers (*n* = 146)	Occasional smokers (*n* = 169)	Regular smokers (*n* = 407)	*p*-value
Gender^a^					**0.002**
Men	312	**39 (12.5)**	**37 (11.9)**	**111 (35.6)**	
Women	1070	**105 (9.8)**	**130 (12.1)**	**283 (26.4)**	
Class^a^					0.137
1st	430	30 (7.0)	60 (14.0)	122 (28.4)	
2nd	461	62 (13.4)	50 (10.8)	118 (25.6)	
3rd	512	54 (10.5)	58 (11.3)	166 (32.4)	
Centre					0.430
Verona	549	48 (8.7)	65 (11.8)	168 (30.6)	
Vicenza	236	21 (8.9)	30 (12.7)	69 (29.2)	
Legnago	197	24 (12.2)	21 (10.7)	59 (29.9)	
Trento	172	21 (12.2)	25 (14.5)	35 (20.3)	
Bolzano	251	32 (12.7)	28 (11.2)	76 (30.3)	

Numbers of past, occasional and regular smokers were reported with percent frequencies in parentheses. *p*-values were computed by Fisher’s exact test for gender, chi-square test for trend for class and chi-square test for centre. Significant results are highlighted in bold

^a^Information on gender and university class was not reported by 25 and 2 students, respectively

**Fig. 1:**
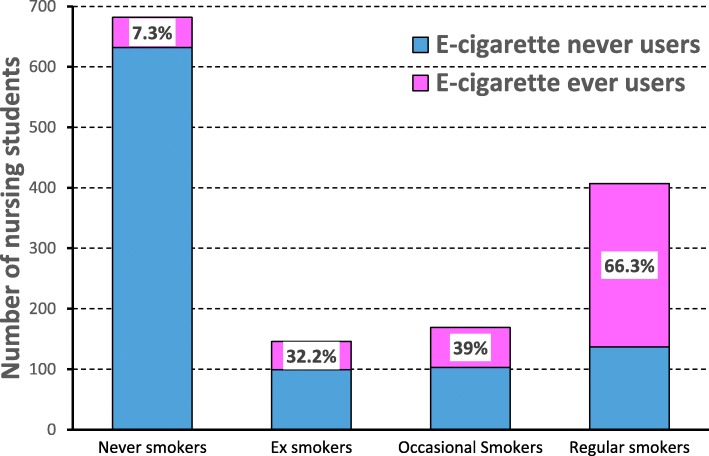
Association between ever use of e-cigarette and tobacco smoking habits

A strong association between tobacco smoking and e-cigarette use was observed. In particular, ever use of e-cigarettes was reported by 57.2% (338/591) of current tobacco smokers and by 12.0% (102/852) of the other students (*p* < 0.001). In detail, the proportion of “e-cigarette ever use” was as low as 7.3% (50/682) among never smokers, increased to 32.2% (47/146) among ex-smokers and 39.0% (66/169) among occasional smokers, and peaked to 66.3% (270/407) among regular smokers (chi-square for trend: *p* < 0.001) (Fig. 1).

Although the use of e-cigarettes in the last month was 10-fold more rare than ever use in the lifespan, it presented a similar pattern with respect to tobacco smoking, being reported by 0.6% (5/851) of non-smokers and by 4.4% (26/584) of current smokers (*p* < 0.001). In detail, e-cigarette current use was nearly absent among never tobacco smokers, being reported by just one subject out of 682 (0.2%); became detectable among ex-smokers (4/146 = 2.7%) and occasional smokers (3/167 = 1.8%); and peaked to 5.5% (22/402) among regular smokers (chi-square for trend: *p* < 0.001).

In multivariable analysis, the odds of e-cigarette ever use significantly increased with incremental use of tobacco smoking: compared to never tobacco smokers, the ORs of ever using e-cigarettes were 5.97 (95% CI 3.46–10.33), 7.75 (5.07–11.85), and 24.6 (16.62–36.50) for ex-smokers, occasional smokers and regular smokers, respectively. The odds of ever using e-cigarettes were more than doubled (OR = 2.54, 95% CI 1.15–5.59) for students living with housemates who used e-cigarettes with respect to those for students whose housemates smoked neither tobacco nor e-cigarettes. Females, as well as students living in centres located in Alpine valleys, had significantly lower odds of being e-cigarette ever users (Table 4).

**Table 4:** Factors associated with ever using e-cigarettes among nursing students of Verona University

Risk factors	OR of e-cigarette ever use (95% CI)	*p-*value
Smoking Habits		
*Never Smokers*	1	
*Ex-Smokers*	**5.97 (3.46–10.33)**	**< 0.001**
*Occasional Smokers*	**7.75 (5.07–11.85)**	**< 0.001**
*Regular Smoker*	**24.6** (**16.62–36.50**)	**< 0.001**
Gender		
*Male*	1	
*Female*	**0.45 (0.32–0.63)**	**< 0.001**
Family history of smoking		
*None*	1	
*Relative who smoked e-cigarettes*	0.68 (0.37–1.25)	0.219
*Relative who smoked tobacco*	1.19 (0.92–1.53)	0.178
Housemates currently smoking		
*None*	1	
*Housemates smoking e-cigarettes*	**2.54 (1.15–5.59)**	**0.021**
*Housemates smoking tobacco*	1.16 (0.94–1.43)	0.163
Centre		
*Verona*	1	
*Vicenza*	**1.12 (1.01–1.24)**	**0.027**
*Legnago*	**0.89 (0.85–0.94)**	**< 0.001**
*Trento*	**0.82 (0.73–0.91)**	**< 0.001**
*Bolzano*	**0.66** (**0.63–0.69**)	**< 0.001**
University class		
1st year	1	
2nd year	1.01 (0.73–1.41)	0.939
3rd year	0.79 (0.56–1.11)	0.173

*OR* odds ratio, 95% CI, and *p*-values were computed by a logistic regression model. Significant results are highlighted in bold

In the multivariable analyses focused on either ever or former smoking, e-cigarette ever use emerged as the strongest predictor among all factors considered. Compared to never users, students who ever used e-cigarettes had 13 times greater odds of being an ever tobacco smoker, whereas they had three times lower odds of being a former smoker (Table 5). Moreover, the odds of being an ever smoker were not affected by family history of smoking but were significantly higher among students living with currently smoking housemates. The odds of having quit smoking among ever smokers tended to increase with increasing university class and were the highest in Legnago, a centre with well-established long-lasting antismoking interventions (Table 5).

**Table 5:** Association between e-cigarette ever-use and ever/former smoking. Ever smoking was evaluated in the whole sample, while former smoking was evaluated among ever smokers

Risk factors	Ever smoking		Former smoking	
	OR (95% CI)	*p*-value	OR (95% CI)	*p*-value
E-cigarette: ever vs never users	**13.45 (9.26–19.55)**	**< 0.001**	**0.33 (0.20–0.53)**	**< 0.001**
Gender (women vs men)	0.90 (0.76–1.06)	0.215	0.73 (0.44–1.22)	0.235
Family history of smoking				
None	1		1	
Users of e-cigarettes	1.32 (0.89–1.95)	0.162	1.05 (0.69–1.60)	0.820
Users of tobacco cigarettes	0.93 (0.73–1.19)	0.562	1.20 (0.58–2.50)	0.617
Housemates currently smoking				
None	1		**1**	
Users of *e-cigarettes*	0.99 (0.72–1.37)	0.975	0.87 (0.62–1.21)	0.416
Users of *tobacco cigarettes*	**2.55 (1.66–3.91)**	**< 0.001**	0.55 (0.26–1.16)	0.116
Centre				
Verona	1		1	
Vicenza	1.02 (0.99–1.05)	0.200	0.95 (0.86–1.05)	0.331
Legnago	**1.11 (1.08–1.14)**	**< 0.001**	**1.44 (1.27–1.63)**	**< 0.001**
Trento	0.97 (0.91–1.04)	0.423	**1.29 (1.03–1.62)**	**0.028**
Bolzano	**1.41 (1.37–1.45)**	**< 0.001**	**1.38 (1.20–1.59)**	**< 0.001**
University class				
1st year	1		**1**	
2nd year	1.12 (0.92–1.37)	0.269	**2.31 (1.04–5.11)**	**0.039**
3rd year	1.35 (0.97–1.86)	0.072	1.47 (0.76–2.86)	0.254

ORs (95% CI) and *p*-values were computed by logistic regression models, controlling for centre, gender, family history of smoking habits, smoking habits among current housemates, and university class. Significant results are highlighted in bold

Seventeen of 26 dual users (students declaring to currently use both electronic and tobacco cigarettes) provided their motivations to use e-cigarettes: tobacco cessation, a decrease in tobacco consumption and a reduction in harmful health effects were reported by 11 (65%), 3 (18%) and 3 (18%) nursing students, respectively. Of note, only three students reported that they had completely stopped smoking thanks to e-cigarette use, and only one of these students was a current vaper.

## Discussion

As shown by the present study performed at the Nursing School of Verona and nearby towns, one-third of the students had ever tried e-cigarettes, but very few students (2.1%) had become regular vapers. In contrast, nursing students presented a rather high prevalence of current tobacco smokers that was slightly higher than the prevalence recorded in the general Verona population of the same age [10] but remarkably higher than the prevalence observed in Italian physicians specializing in Public Health [24]. E-cigarette ever use was cross-sectionally associated with a higher risk of starting smoking and with a lower risk of quitting smoking. Of note, only a trivial proportion of nursing students reported that they had stopped smoking thanks to e-cigarette use.

### Prevalence of users of e-cigarettes and conventional cigarettes

Awareness of e-cigarettes was nearly identical in the present survey (94.7%) and in a national sample of the same age (15–24 years) (94.8%) [12]. Similarly, the prevalence of current e-cigarette users in the present survey and in the national sample was 2.1 and 2.4%, respectively. In contrast, the prevalence of ever use of e-cigarettes was three times higher in nursing students from north-eastern Italy (30.3%) than in the national sample of individuals who were in the same age range (15–24 years) (11.6%) [12]. It should be noted, however, that the present survey considered the life span, while the national survey considered only the previous 2 years. In the present study, both ever use and current use of e-cigarettes were higher among men (43.8 and 3.7%, respectively) than women (26.3 and 1.7%, respectively), in agreement with the current literature [12].

The proportion of e-cigarette ever use reported by Italian nursing students (30.3%) was comparable to the proportion recorded in other academic settings, which ranged from 24 to 28% among American college students [21, 25] to 36% among French military nursing students [19]. Regarding current e-cigarette use, vapers were rare both among responders in the present study (2.1%) and among medical students from New York (1.6%) [20], while a much larger proportion was recorded among French military nursing students (25%) [19] and Arkansas health professional students (20.6%) [21].

Regarding tobacco smoking, the proportion of regular smokers among nursing students from north-eastern Italy (35.6% of men and 26.4% of women) was slightly higher than the proportion of current smokers among men and women from the same area (32.9 and 23.5%, respectively) [10]. It should be noted that the definition of current smoking slightly differed between the two surveys: it was defined as “having smoked several times per week in the last 30 days” in the present survey and as “having smoked at least one cigarette per day per one year and also in the last month” in the general population study [10]. Of note, a lower proportion of current smokers was recorded in 2015 in the general Italian population: 21.3, 27.6, and 33.5% of men and 16.4, 19.9, and 19.1% of women aged 18–19, 20–24, and 25–34 years, respectively [26].

On the other hand, the prevalence of tobacco smoking recorded among students in the present survey was similar to that previously recorded among nursing students in Europe (34.3% of men and 27.5% of women) [27] and Greece (30.5% of men and 30.6% of women) [28] and in health professional students in the US (27.8% overall) [21]. A larger prevalence (40%) was recorded for French military nursing students and instructors [19]. The prevalence was lower among medical students from New York (3.9% smoking cigarettes and 2.1% smoking cigars) [20], among a population of mostly undergraduate students from Midwestern University (16.3%) [25] and among Italian post-graduate students attending the School of Public Health (26.2% of men and 16.2% of women) [24]. Current smoking was particularly rare among Chinese medical/health professional students, with an overall prevalence of only 7% [29].

In addition, e-cigarette ever use, as well as conventional smoking initiation, was associated with housemates’ smoking habits but not with a family history of smoking. A pro e-cigarette social environment, such as housemates currently smoking e-cigarettes, represented a very important incentive to start using e-cigarettes, in agreement with the current literature [30, 31]. Conversely, the odds of smoking initiation were more than doubled among people living with current smoking housemates.

Although nursing students of north-eastern Italy had been trained about risk factors and health promotion, they did not present a healthier lifestyle with respect to their contemporaries from the general population, with a higher proportion of both current smokers and e-cigarette ever users among these students. Of note, the prevalence of tobacco smoking tended to decrease from the first class to subsequent classes; moreover, the odds of being a former smoker were the highest in the centre (Legnago) with active antismoking interventions. This finding suggests that the knowledge acquired within the nursing curriculum may improve awareness of risk factors and promote a healthy lifestyle.

### Relation between conventional tobacco smoking and e-cigarette use

E-cigarette and tobacco smoking were strongly related in the present study of Italian nursing students, in agreement with the current literature. Indeed, the prevalence of e-cigarette ever use as a function of smoking habits (7.3, 32.2 and 57.2% among never, past and current tobacco smokers, respectively) was similar to values found among French military nursing students (21.9 and 57% among non-smokers and current tobacco smokers, respectively) [19] and among American college students (13.9, 45, 74.2% among never, past and current tobacco smokers, respectively) [25].

A fundamental public health question is if e-cigarette use could be a gateway to nicotine addiction and consequently to tobacco smoking initiation. The cross-sectional design of the present study does not allow the cause-effect relation between e-cigarette use and tobacco smoking to be addressed. While taking this limitation into account, the present study noted that e-cigarette ever users had higher odds of being ever smokers than did never users. This association was particularly strong, with an OR of 13.5 for having smoked tobacco at least sometimes. Accordingly, many studies, from meta-analyses to longitudinal and cross-sectional studies, support the notion that e-cigarette use among adolescents/young adults increased the probability for future use of traditional cigarettes [16, 30, 32].

Regarding smoking cessation, the odds of being a former smoker among ever smokers were three times lower for e-cigarette ever users than for never users (OR = 0.33). This finding was supported by a longitudinal study on current smokers enrolled in American colleges, where trying e-cigarettes more than doubled the risk of retaining smoking habits after a three-year follow-up [31]. Furthermore, a survey on French university students found that even if 12.6% of cigarette ever smokers had given up smoking thanks to e-cigarette use, a higher proportion (20%) had begun to smoke conventional cigarettes after using e-cigarettes [33].

### Limitations

Some limitations should be acknowledged. First, although most students attending the nursing school in the 5 different centres answered the questionnaire, the present sample was not representative of the Italian population of nursing students. Second, the low number of students reporting current use of e-cigarettes (*n* = 31) limited the possibility of evaluating the determinants of this condition. Third, the cross-sectional design did not allow investigation of the direction of the association between e-cigarette use and conventional tobacco smoking. Finally, smoking habits were assessed by questionnaire; however, a good agreement (Cohen’s k = 0.93) between self-reported smoking consumption and serum cotinine levels was found among young adults of Verona [34].

## Conclusions

The use of e-cigarettes seemed to be rather rare among Italian nursing students and was mainly restricted to current smokers. One-third of students had ever tried e-cigarettes, but only 2% had become regular vapers. E-cigarette ever use was as low as 7% among never smokers and peaked to 66% among regular smokers. E-cigarette use did not appear to be an instrument to stop smoking among nursing students. Moreover, the present study also highlights the importance of school-based prevention programmes to correct respiratory health habits, given the alarming high prevalence (40.9%) of tobacco smoking among nursing students. Indeed, health professional students will play an essential role in promoting healthy habits and counselling the general population about smoking cessation.

## Additional files

Additional file 1: Questionnaire, English version. (PDF 351 kb)

Additional file 2: Dataset. (XLS 382 kb)

## Data Availability

The datasets generated and/or analysed during the current study are available as Additional file 2.

## Abbreviations

ERS: European Respiratory Society
ETINCEL-OFDT: “Enquête téléphonique pour l’information sur la cigarette électronique” of the “Observatoire français des drogues et des toxicomanies”
GRADE: Grading of Recommendations Assessment, Development and Evaluation
OR: odds ratio
