# Supplementary material for: Knowledge and use of e-cigarettes among nursing students: results from a cross-sectional survey in north-eastern Italy
Source: BMC Public Health. 2019 Jul 22;19:976. doi: 10.1186/s12889-019-7250-y (PMC6647064; doi:10.1186/s12889-019-7250-y)
Supplement: Supplementary file 1 — Questionnaire, English version. (PDF 351 kb) [file 12889_2019_7250_MOESM1_ESM.pdf]

E-CIGARETTES SURVEY

The present questionnaire is an English translation of a modified Italian version of the ETINCEL (Telephone survey for information about electronic cigarette) questionnaire, developed in French by OFDT (*Observatoire français des drogues et des toxicomanies*).

Class in the nursing school: I- II- III Campus: Verona | Vicenza | Legnago | Trento | Bolzano

Year of birth: _ _ _ _ Gender: M | F

Have you ever lived with smoking relatives? ☐ Yes, e-cigarette ☐ Yes, tobacco ☐ no

Are you living with people who smoke? ☐ Yes, e-cigarette ☐ Yes, tobacco ☐ no

I PART: E-cigarettes

1) Have you ever heard about "electronic cigarette", also called as "e-cig" or "personal vaporizer"? SÌ NO

2) Have you ever tried an electronic cigarette? SÌ NO

If you answered "no" to question 2:

3) Are you going to try an electronic cigarette in the future? SÌ NO

If you answered "yes" to question 2:

4) Have you been smoking electronic cigarettes in the last 30 days (not considering the first time you ever used it)? SÌ NO

If you answered "no" go to question n.10

5) How often do you use electronic cigarettes?
   - ☐ Every day
   - ☐ Several times a week
   - ☐ Once a week
   - ☐ Two/three times per month
   - ☐ Once a month
   - ☐ I don't know

6) When did you start using electronic cigarettes?
   - ☐ During the last 30 days
   - ☐ More than one month ago but less than 6 months ago
   - ☐ More than 6 months ago but less than 1 year ago
   - ☐ More than 1 year ago
   - ☐ I don't know

6.1) Do you have your own electronic cigarette?

- Yes, I have mine
- No, I share it with another person
- No, I borrow it from other people
- Other (Specify) ………

7) Where have you bought your own e-cigarette last time?

- In a shop specialized in electronic cigarettes
- On the internet
- In a tobacco shop
- At the chemist's
- Other (specify) ……

8) Where have you bought the refill (e-liquid, cartridge) for your electronic cigarette last time?

- In a shop specialized in electronic cigarettes
- On the internet
- In a tobacco shop
- At the chemist's
- Other (specify)
- Not pertinent, I use disposable e-cigarettes

9) Which is the nicotine dose of the electronic cigarette you usually smoke? _____________
(If your device is nicotine-free, write 0. If you don't know the dose, write 99)

II PART: Tobacco

The next questions refer to your tobacco use (cigarettes, rolling tobacco, cigars, cigarillos, pipe, chicha/narguilè,…) apart from your e-cigarettes use.

10) Do you smoke, even occasionally, tobacco? SÌ NO

If you answered "yes" to question 10

11) How often have you been smoking tobacco in the last 30 days?

- Every day
- Several times a week
- Once a week
- Two-three times a month
- Only once a month
- I don't know

If you answered "no" to question 10:

12) Have you ever smoked tobacco (either regularly or occasionally) in your life?

- Yes, regularly
- Yes, sometimes
- I've only tried it
- Never

If you answered "yes" to questions 12 and 4:

13) Did you completely stop smoking tobacco thanks to e-cigarettes? SÌ NO

If you answered "yes" to question 10:

14) Where did you buy tobacco (cigarette pack, rolling tobacco, pipe tobacco, cigar) for your personal use last time?

- In a tobacco shop in Italy
- In a tobacco shop in a neighbouring country (France, Swiss, Austria, Slovenia)
- In a tobacco shop in another Country
- In a duty-free
- On the internet
- On the street
- Other
- I don't know

III PART: Tobacco and e-cigarette

If you answered “yes” to questions 4 and 10:

15) You are actually using….?

- ☐ More often e-cigarettes and sometimes tobacco
- ☐ E-cigarettes and tobacco with the same frequency
- ☐ More often tobacco and sometimes e-cigarettes
- ☐ Other

If you answered “yes” to questions 4 and 10:

16) Which is the main reason why you use e-cigarette?

- ☐ To completely stop smoking (both tobacco and e-cigarettes)
- ☐ To reduce my tobacco consumption, without completely stopping smoking tobacco at the moment
- ☐ To reduce my tobacco consumption , without stopping smoking e-cigarettes at the moment
- ☐ To replace tobacco use with e-cigarette use
- ☐ To reduce the amount of money spent for tobacco/ to save money
- ☐ To have the opportunity to inhale vaping wherever I want (at office, at home…)
- ☐ To reduce troubles related to tobacco use (bad breath, yellow teeth, tobacco smell)
- ☐ To reduce health risks without stoppingsmoking tobacco / It is less dangerous for my health
- ☐ To stop being nicotine addicted/ to progressively reduce my nicotine addiction by using e-cigarettes
- ☐ Other (specify) ……
- ☐ I don’t know
